# Supplementary material for: Absolute dating of the European Neolithic using the 5259 BC rapid 14C excursion
Source: Nat Commun. 2024 May 20;15:4263. doi: 10.1038/s41467-024-48402-1 (PMC11106086; doi:10.1038/s41467-024-48402-1)
Supplement: Supplementary file 1 — Supplementary Information [file 41467_2024_48402_MOESM1_ESM.pdf]

Supplementary Information

for “Absolute dating of the European Neolithic using the 5259 BC rapid 14C excursion”, Maczkowski, Francuz, Pearson, Giagkoulis, Bolliger, Szidat, Wacker, Kotsakis & Hafner, 2024, Supplementary Information DOI: 10.5281/zenodo.8407222

Supplementary Note 1

Supplementary Note 1.1

Dispilio oak chronology wiggle-matching OxCal CQL code, results, and dates list

The oak chronology from Dispilio (with chronology ID code 6001) has a robust dendrochronological cross-dating against the juniper chronology over a period of 108 years where wood sample replication is >4 (t-value$_{HO}$= 4.9[1], and t-value$_{BP}$= 5.1[2]; GLK = 63%[3]). This dendrochronological placement is further supported by a conventional radiocarbon wiggle-matching model presented below (Supplementary Figure 1.1 and Supplementary Figure 1.2). The wiggle-matching model was constructed with radiocarbon measurements obtained from tree-rings sampled from oak wood samples comprising the oak 6001 tree-ring width chronology. Tree-ring sampling for radiocarbon measurements was performed in 2019, before the construction of the juniper tree-ring chronology[4]. Tree-rings were sampled from 8 different oak wood samples (Supplementary Table 1.1 below) whose tree-ring width sequences make up the oak tree-ring width chronology. Access to OxCal online: https://c14.arch.ox.ac.uk/oxcal/OxCal.html# (accessed 16.04.2024)

#OxCal CQL code:

```
Options()
 {
  Resolution=1;
 };
 Plot()
 {
  D_Sequence("Disp-6001 QUSP")
  {
   R_Date("BE-12206.1.1 R.3-12", 6491, 24);
   Gap(1);
   R_Date("BE-12197.1.1 R.6-10", 6444, 23);
   Gap(4);
```

```
  R_Date("BE-12220.1.2 R.10-14", 6484, 28);
  Gap(31);
  R_Date("BE-12222.1.1 R.41-45", 6448, 23);
  Gap(9);
  R_Date("BE-12204.1.1 R.51-54", 6415, 24);
  Gap(16);
  R_Date("BE-12215.1.1 R.67-70", 6398, 23);
  Gap(1);
  R_Date("BE-12207.1.1 R.65-73", 6406, 24);
  Gap(14);
  R_Date("BE-12214.1.1 R.80-86", 6397, 23);
  Gap(26);
  R_Date("BE-12205.1.1 R.104-114", 6359, 24);
  Gap(0);
  R_Date("BE-12216.1.1 R.106-112", 6311, 24);
  Gap(5);
  R_Date("BE-12223.1.1 R.110-120", 6316, 23);
  Gap(1);
  Date("modelled end-date Dispilio oak chrono");
 };
};
```

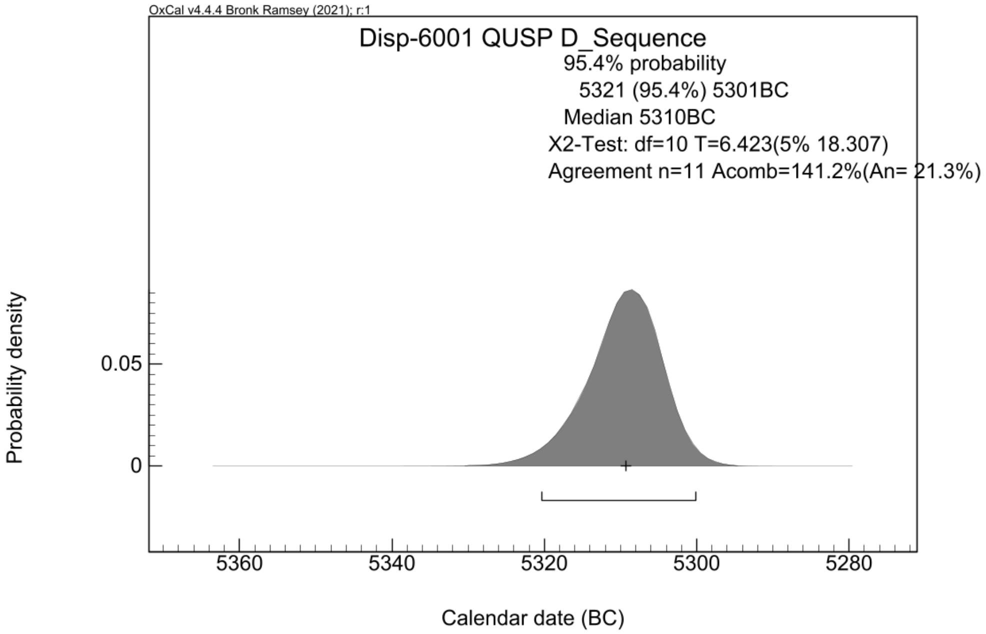

Supplementary Figure 1.1 *Dispilio oak chronology modelled end-date range through wiggle-matching in OxCal 4.4* [5,6]

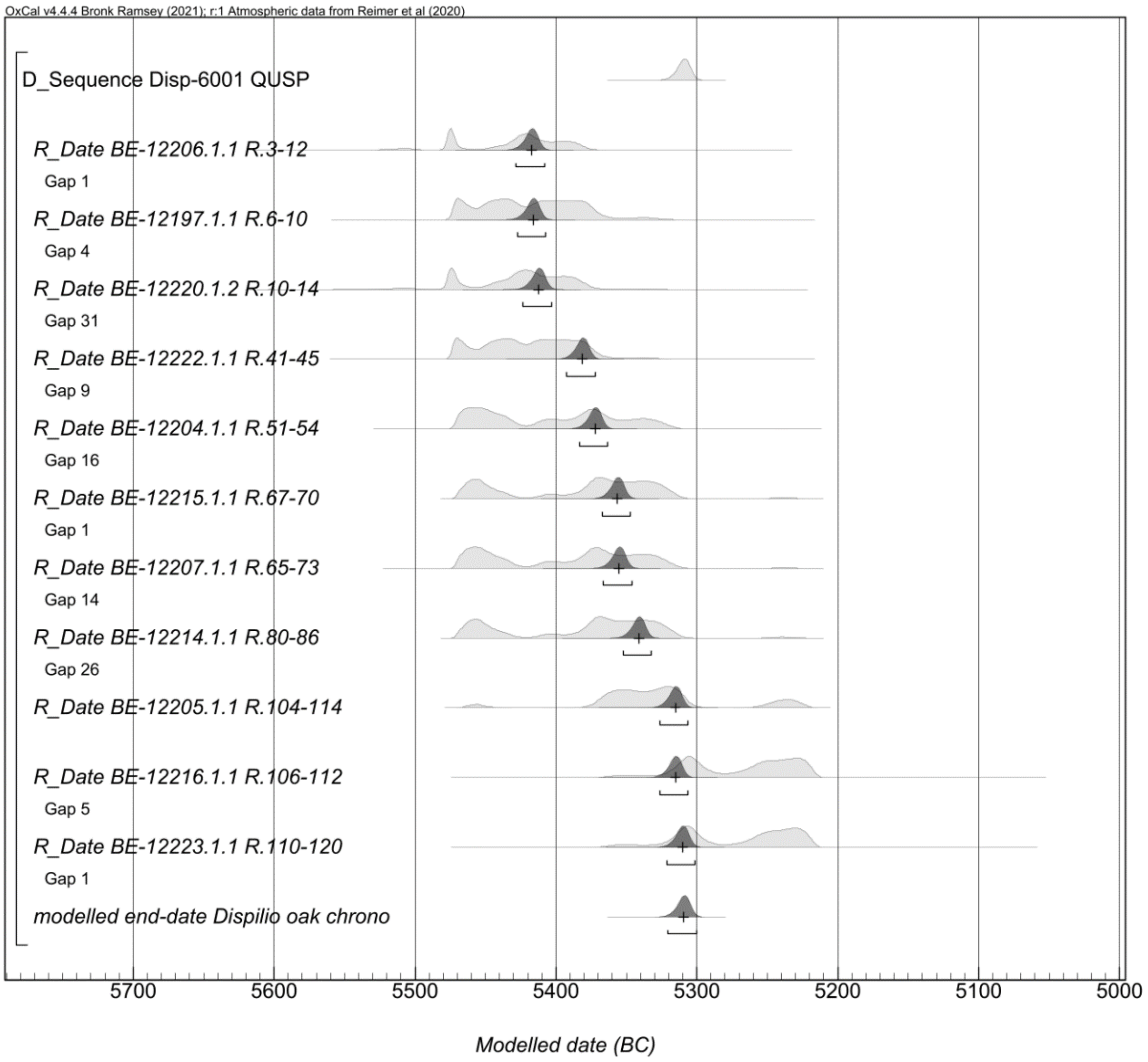

Supplementary Figure 1.2 *Dispilio oak chronology wiggle-matching, same as Supplementary Figure 1.1, but including the probability distribution for each ^{14}C date that makes up the model; OxCal 4.4* [5,6]

14C-Lab Code	14C Sample Code	Wood sample excav ID	Species	Tot. no. of rings	Rings sampled for 14C	Chronology ID	Rings on chronology	Median ring on chrono	Age uncal (y BP)	±1s (y)
BE-12206.1.1	DISP_10737_01	DISP-10737	*Quercus* spp.	107	3-12	DISP-6001	3-12	7	6491	24
BE-12197.1.1	DISP_10427	DISP-10427	*Quercus* spp.	63	3-7	DISP-6001	6-10	8	6444	23
BE-12220.1.2	DISP_10835	DISP-10835	*Quercus* spp.	105	4-8	DISP-6001	10-14	12	6484	28
BE-12222.1.1	DISP_10886	DISP-10886	*Quercus* spp.	57	1-5	DISP-6001	41-45	43	6448	23
BE-12204.1.1	DISP_10707_01	DISP-10707	*Quercus* spp.	73	4-7	DISP-6001	51-54	52	6415	24
BE-12215.1.1	DISP_10813_01	DISP-10813	*Quercus* spp.	61	15-18	DISP-6001	67-70	68	6398	23
BE-12207.1.1	DISP_10737_02	DISP-10737	*Quercus* spp.	107	65-73	DISP-6001	65-73	69	6406	24
BE-12214.1.1	DISP_10808	DISP-10808	*Quercus* spp.	52	46-52	DISP-6001	80-86	83	6397	23
BE-12205.1.1	DISP_10707_02	DISP-10707	*Quercus* spp.	73	57-67	DISP-6001	104-114	109	6359	24
BE-12216.1.1	DISP_10813_02	DISP-10813	*Quercus* spp.	61	54-60	DISP-6001	106-112	109	6311	24
BE-12223.1.1	DISP_10899	DISP-10899	*Quercus* spp.	53	43-53	DISP-6001	110-120	114	6316	23

Supplementary Table 1.1 *List of all ^{14}C measurements included in the wiggle-matching model of the oak chronology 6001*

Supplementary Note 1.2

Initial wiggle-matching of the juniper chronology OxCal CQL code, results, and dates list (before the discovery of the Miyake event)

The data below consists of the initial radiocarbon measurements obtained from the juniper chronology to anchor it approximately on the calendar scale. The initial wiggle-matching of these dates produced the modelled end-date range for the chronology. The end-date range at 93.2% probability (5165-5137 cal BC, Supplementary Figure 1.3 below) served as the basis for identifying the approximate area along the chronology where the ^{14}C cosmogenic signature of 5259 BC was to be located. Note the poor OxCal agreement index (Acomb=10.7%; rule of thumb should be >60%), resulting from the absence of the 5259 BC event in the IntCal20 calibration curve. These dates presented below were not used in the analyses presented in the main article text and are now superseded by the new 115 ^{14}C measurements described in the main article text and contained in the Supplementary Data 1 (under the same DOI as this document: 10.5281/zenodo.8407222).

#OxCal CQL code:

```
Options()
 {
 Resolution=1;
 };
 Plot()
 {
  D_Sequence("Disp Juniper Chrono initial")
  {
   R_Date("BE-16932.1.1", 6558, 34);
   Gap (38);
   R_Date("BE-16933.1.1", 6523, 35);
   Gap (35);
   R_Date("BE-16934.1.1", 6463, 34);
   Gap (42);
   R_Date("BE-16935.1.1", 6355, 35);
   Gap (37);
   R_Date ("BE-17771.1.1",6259, 31);
   Gap(11);
   R_Date("BE-16940.1.1", 6349, 34);
   Gap (7);
   R_Date("BE-16936.1.1", 6255, 34);
```

```
  Gap (48);
  R_Date("BE-16937.1.1", 6282, 33);
  Gap (16);
  R_Date("BE-16938.1.1", 6202, 34);
  Gap (30);
  R_Date("BE-17772.1.1",6230, 31);
  Gap (18);
  R_Date("BE-16939.1.1", 6266, 34);
  Gap (3);
 };
};
```

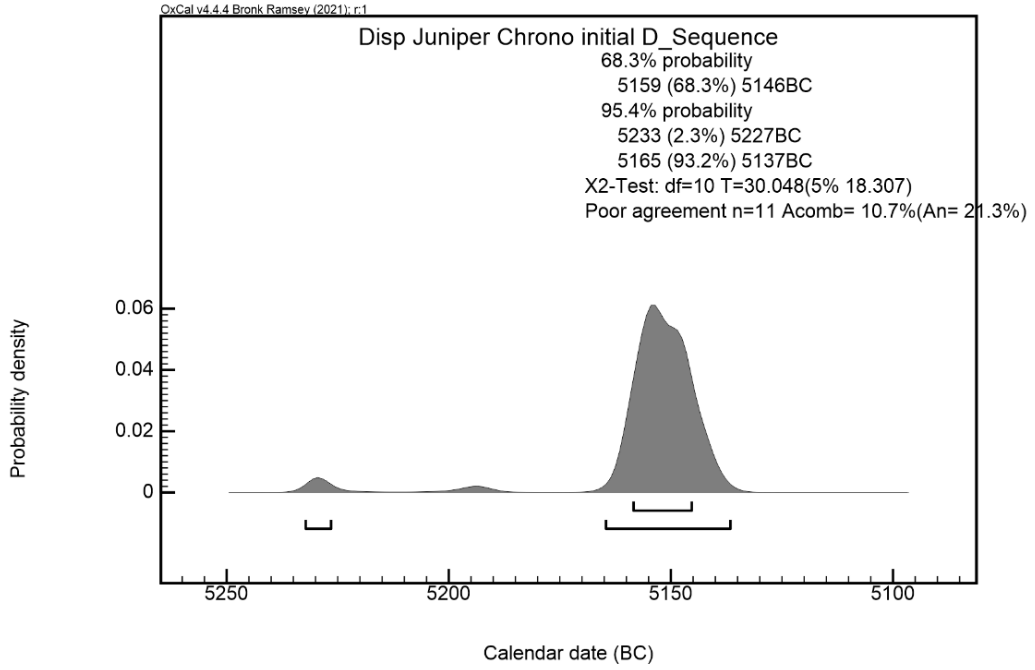

Supplementary Figure 1.3 *Single plot of Dispilio juniper chronology* <u>*initial*</u> *modelled end-date range through wiggle-matching in OxCal 4.4. The end-date range at 93.2% probability served as the basis for identifying the approximate area along the chronology where the ^{14}C cosmogenic signature of 5259 BC was to be located. ^{14}C measurements used to produce this figure were not used in any of the analyses presented and described in the main article text. Note the poor OxCal agreement index (Acomb=10.7%), resulting from the absence of the 5259 BC event in the IntCal20 calibration curve.*

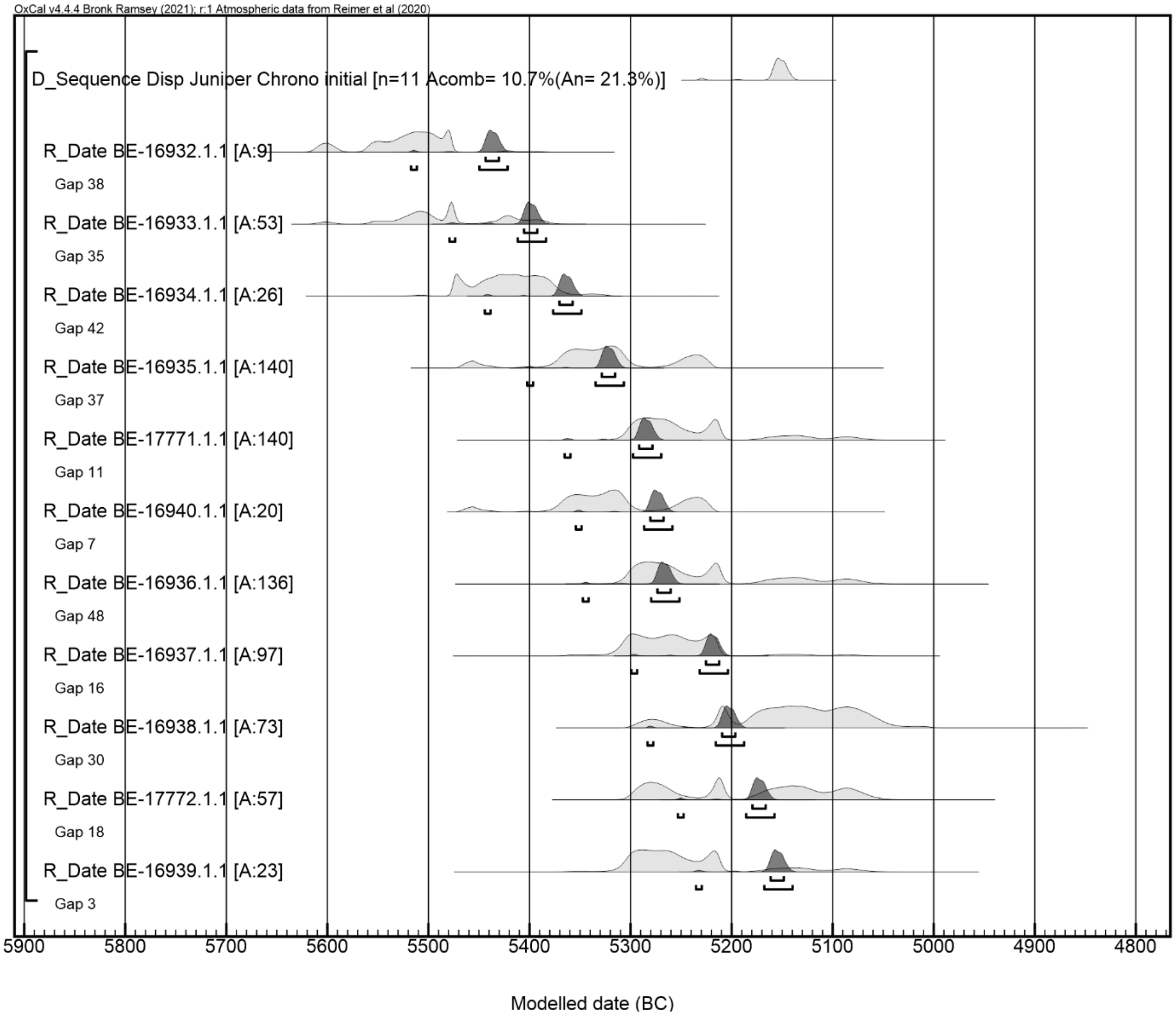

Supplementary Figure 1.4 *Same wiggle-matching model as in previous Supplementary Figure 1.3, but with the posterior likelihoods of all individual dates plotted. Wiggle-matching in OxCal 4.4.*

14C-Lab Code	14C Sample Code	Wood sample excav ID	Species	Tot. no. of rings	Rings sampled for 14C	Chronology ID	Rings on chronology	Median ring on chrono	Age uncal (y BP)	±1s (y)
BE-16932.1.1	DISJ.1.10557.5-7	DISP-10557	*Juniperus* spp.	173	5-7	DISP-401	15-17	16	6559	35
BE-16933.1.1	DISJ.2.1490.18-20	DISP-10490	*Juniperus* spp.	112	18-20	DISP-401	53-55	54	6523	35
BE-16934.1.1	DISJ.3.10509.45-47	DISP-10509	*Juniperus* spp.	104	45-47	DISP-401	88-90	89	6463	34
BE-16935.1.1	DISJ.4.10528.42-44	DISP-10528	*Juniperus* spp.	59	42-44	DISP-401	130-132	131	6355	35
BE-17771.1.1	DISJ.10.10374.12-14	DISP-10374	*Juniperus* spp.	133	12-14	DISP-401	167-169	168	6259	31
BE-16940.1.1	DISJ.6.10062.67-69	DISP-10062	*Juniperus* spp.	90	67-69	DISP-401	178-180	179	6349	34
BE-16936.1.1	DISJ.5.10611.23	DISP-10611	*Juniperus* spp.	123	23	DISP-401	186	186	6256	34
BE-16937.1.1	DISJ.7.10615.18	DISP-10615	*Juniperus* spp.	72	18	DISP-401	234	234	6283	33
BE-16938.1.1	DISJ.8.10615.34	DISP-10615	*Juniperus* spp.	72	34	DISP-401	250	250	6203	34
BE-17772.1.1	DISJ.11.10374.124-26	DISP-10374	*Juniperus* spp.	133	124-26	DISP-401	279-281	280	6230	31
BE-16939.1.1	DISJ.9.10570.76	DISP-10570	*Juniperus* spp.	79	76	DISP-401	298	298	6267	34

Supplementary Table 1.2 *List of the ^{14}C measurements included in the initial wiggle-matching model of the juniper chronology.*

Supplementary Figures

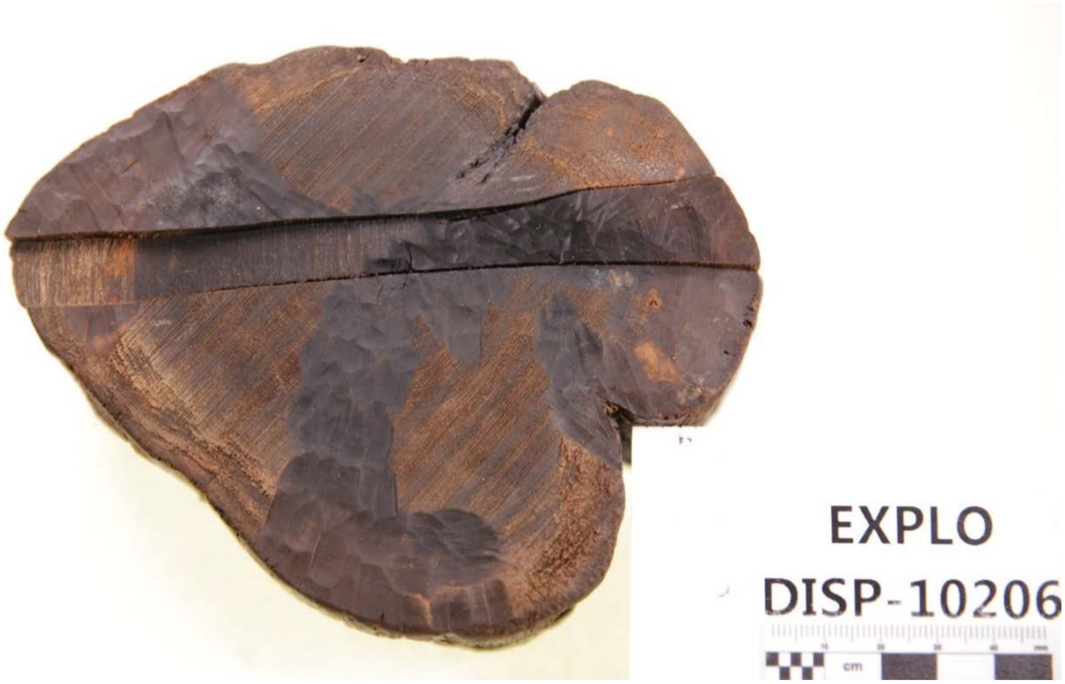

***Supplementary Figure 1** Archaeological wood sample DISP-10206*

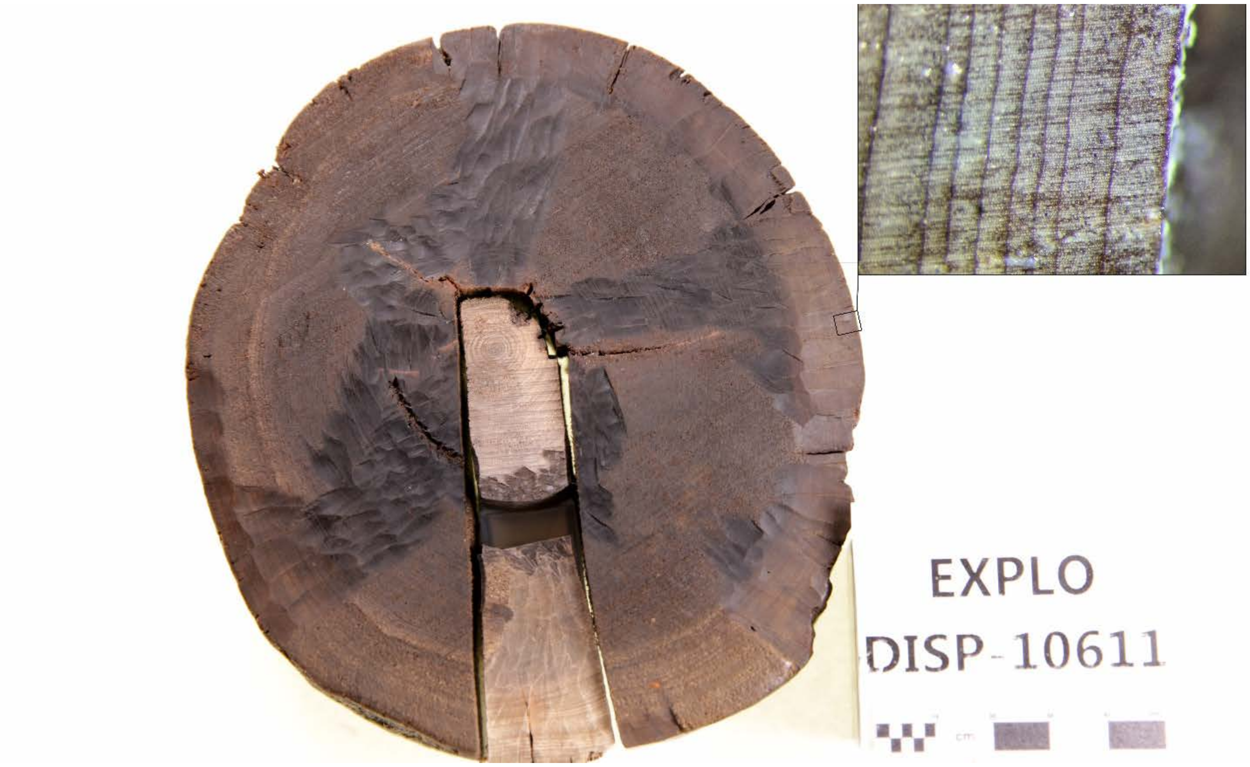

***Supplementary Figure 2** Archaeological wood sample DISP-10611; inset: close-up of the samples' waney-edge (last growth ring), tracheids filled with chalk to enhance contrast.*

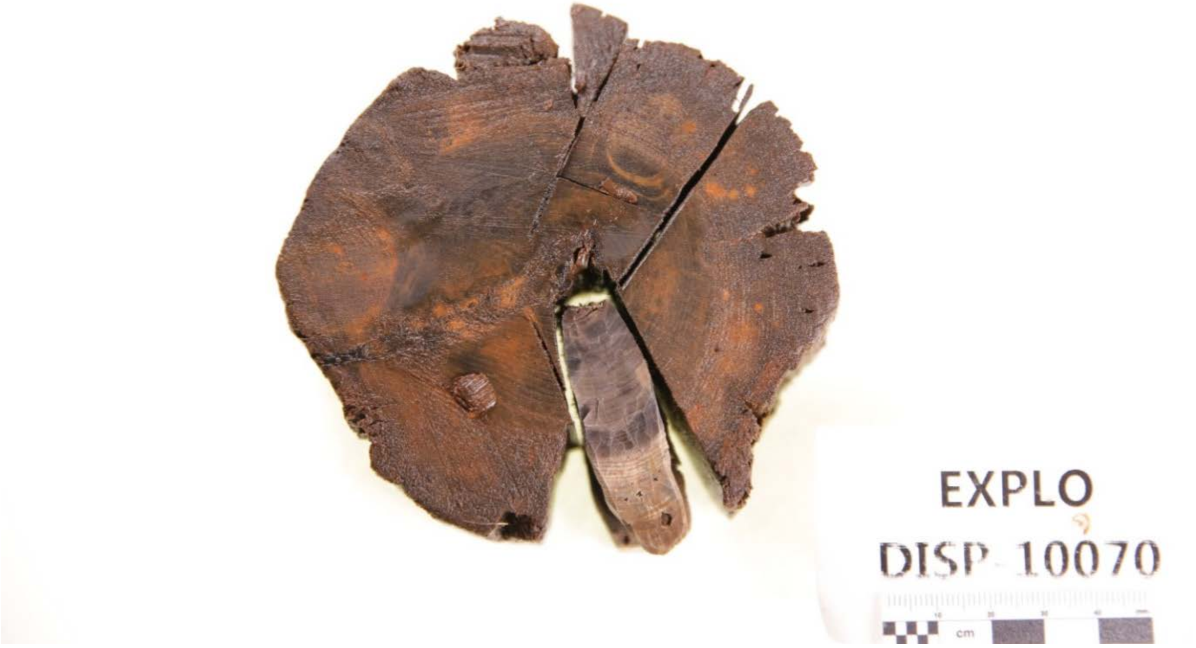

Supplementary Figure 3 *Archaeological wood sample DISP-10070*

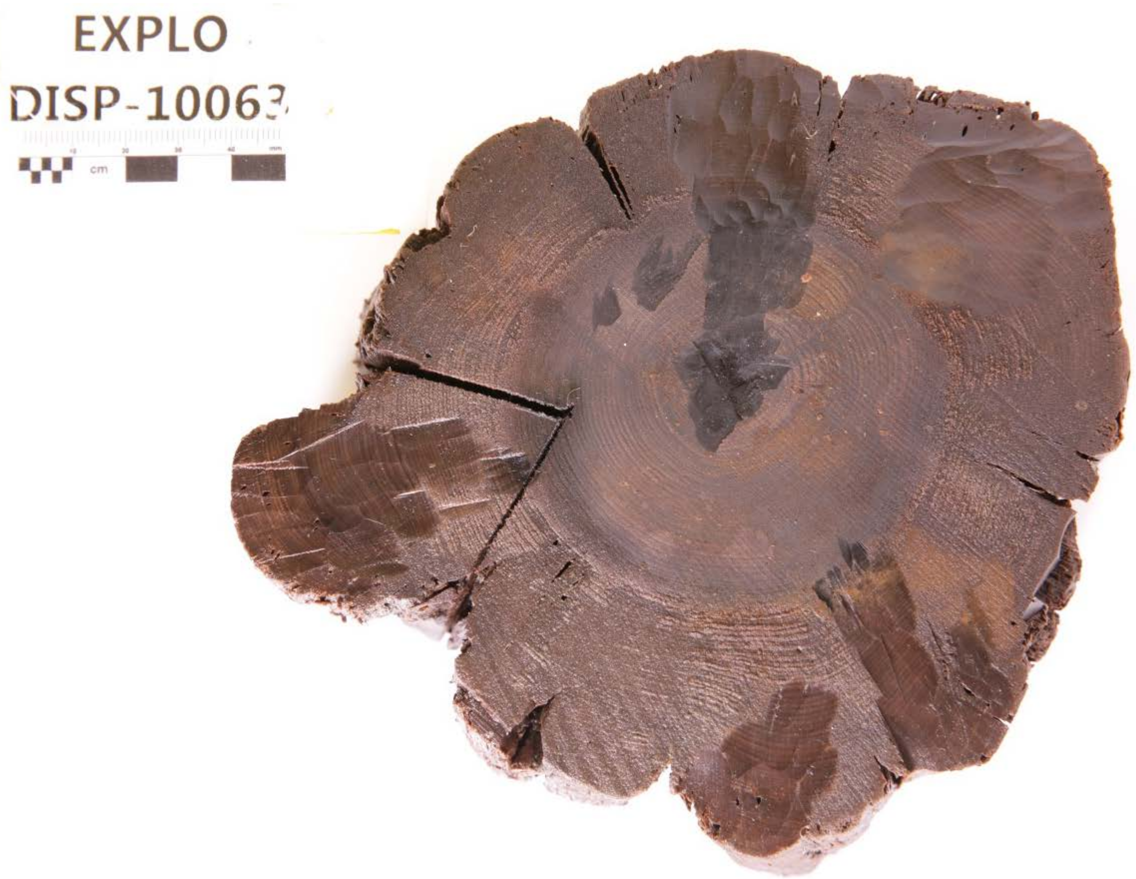

Supplementary Figure 4 *Archaeological wood sample DISP-10063*

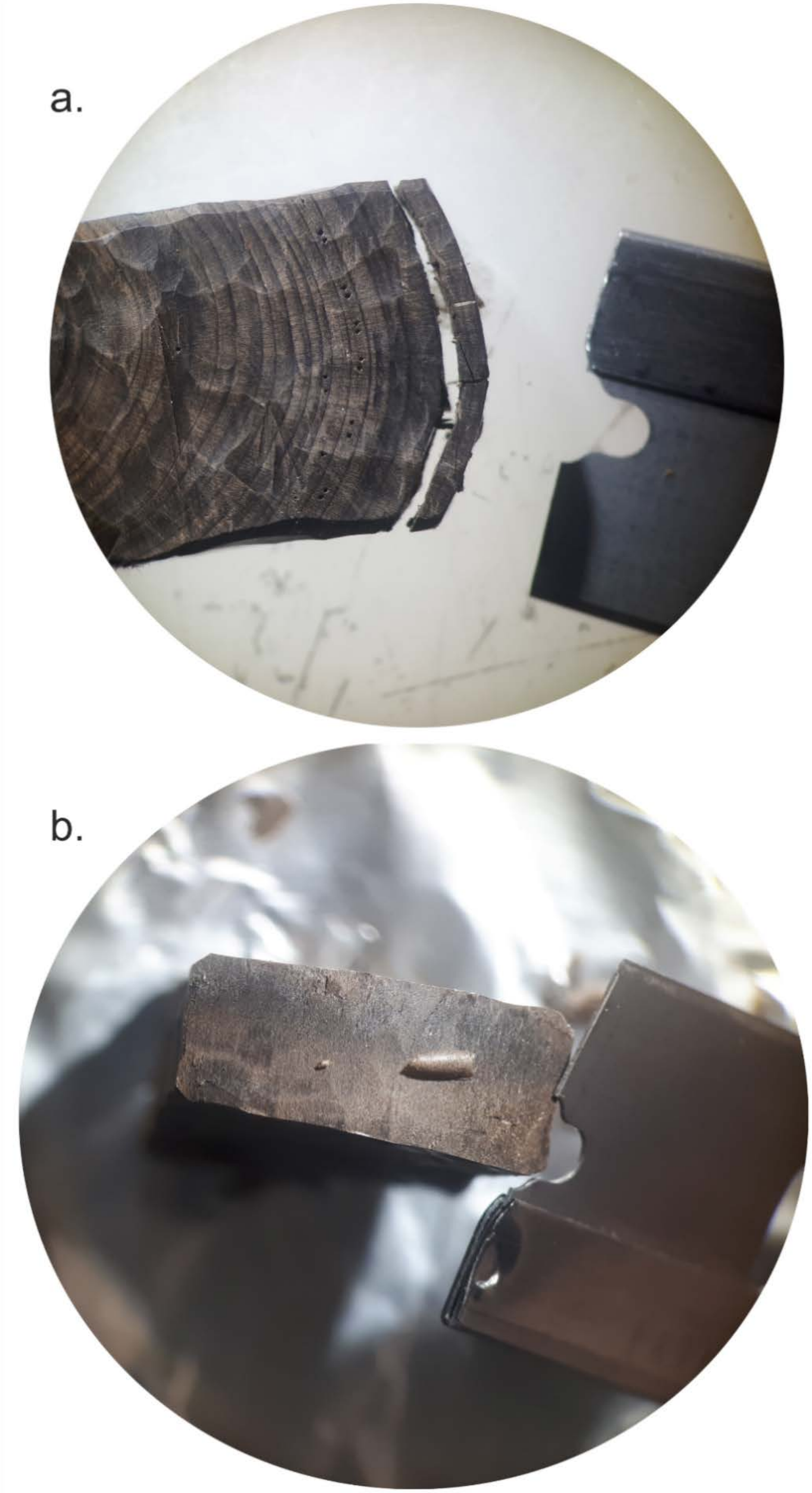

Supplementary Figure 5 *Details from the ring sampling of DISP-10611:* ***a****.: sampling an individual ring. The "event ring" is ring number 19, located just below the ring 20 which is marked with a series of double pins;* ***b****.: scraping off last bits of earlywood, tangetial view of the wood block in a.; width of razor side 1.8 cm.*

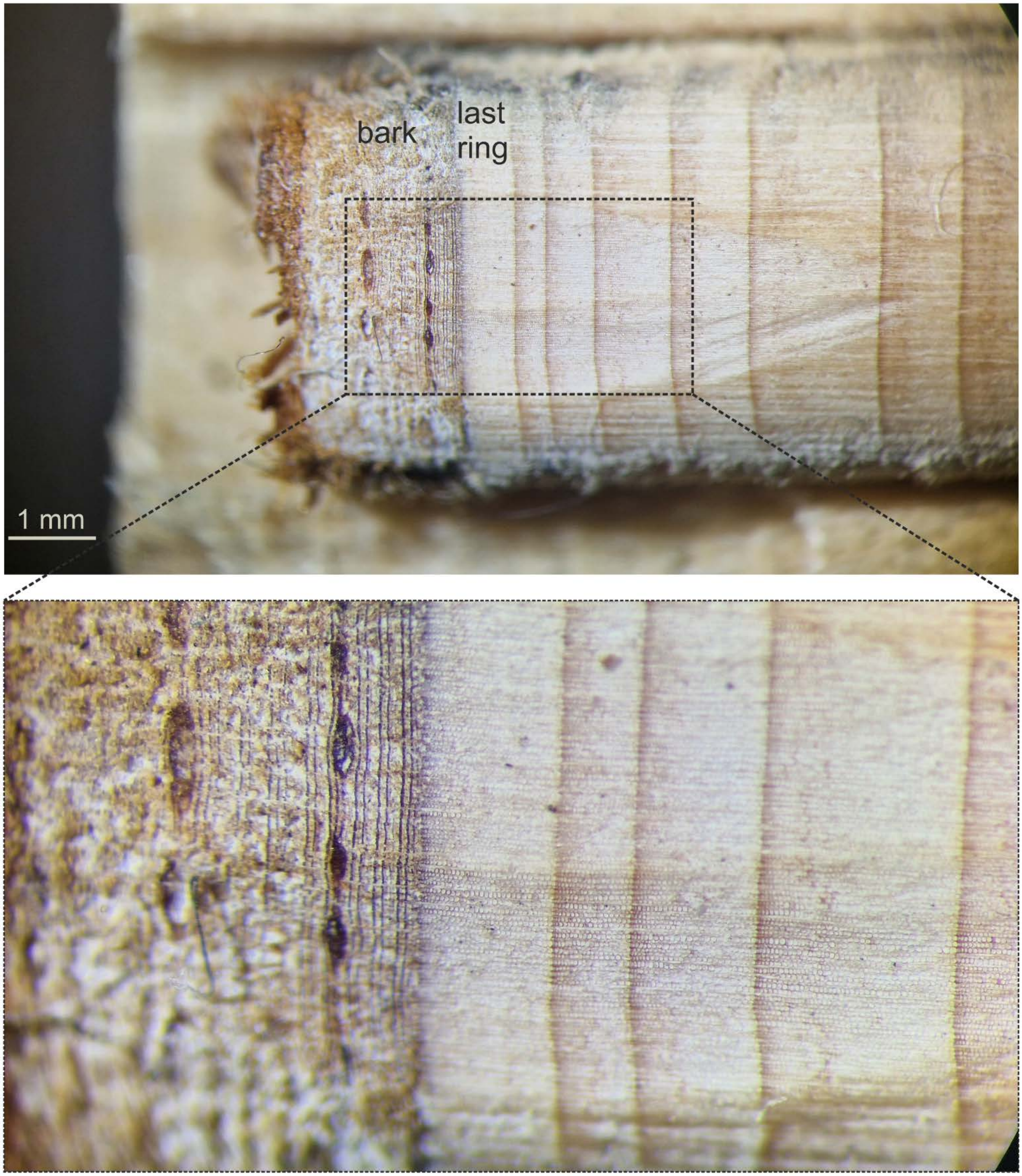

Supplementary Figure 6 *Cross-section of a* *<u>modern</u>* *Juniperus excelsa from Mt. Galichica, ca. 60 km northwest of Dispilio. Latewood of last ring is almost fully developed, same as in the oaks in the area, indicating similar timing of ring formation. Core taken between 5-7 September 2021.*

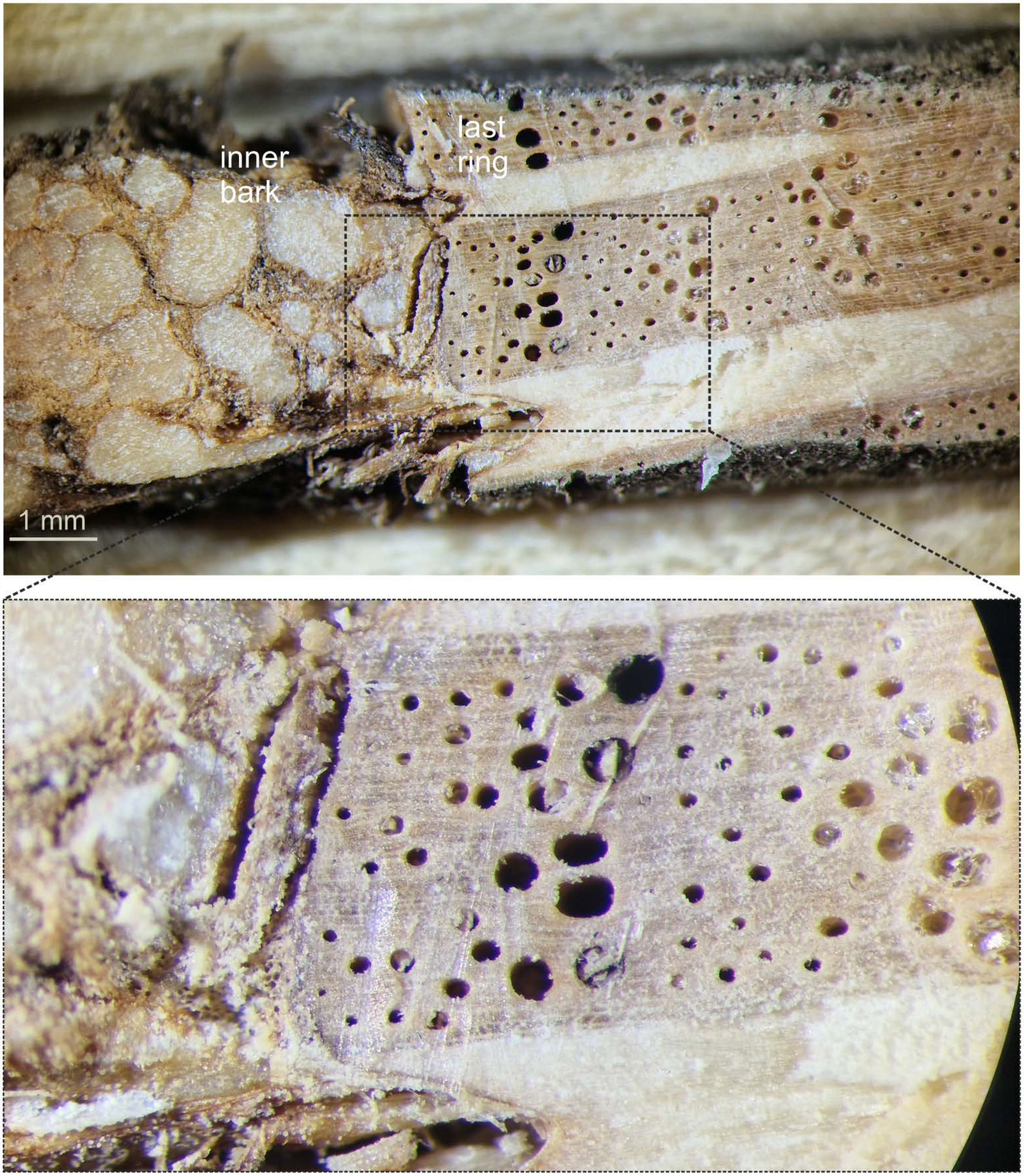

Supplementary Figure 7 *Cross-section of a <u>modern</u> Quercus trojana from Mt. Galichica, ca. 60 km northwest of Dispilio. Latewood of last ring is fully developed, same as in junipers in the area, indicating similar timing of ring formation. Core taken between 5-7 September 2021.*

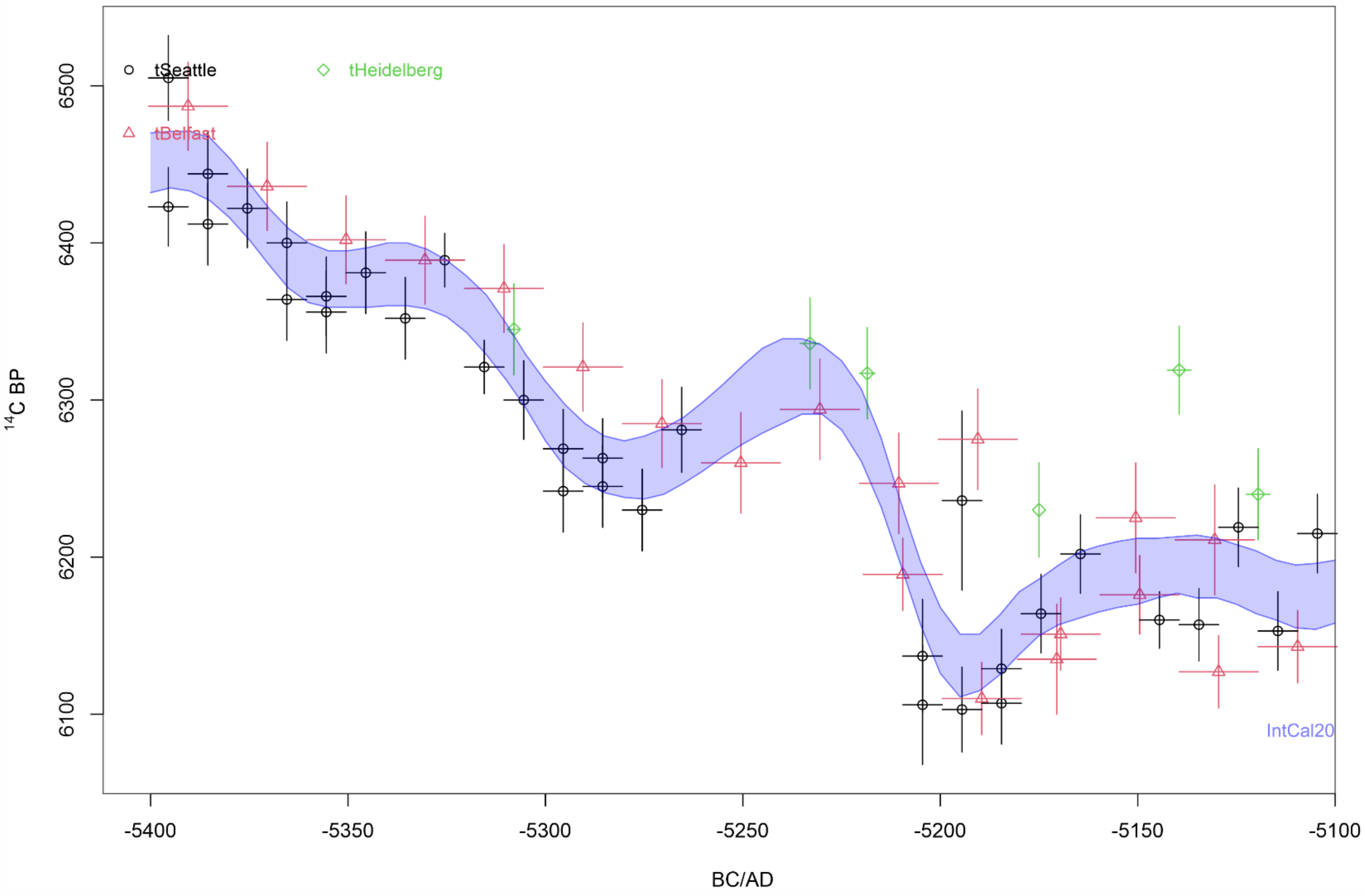

Supplementary Figure 8 *Radiocarbon calibration curve IntCal20 [6] (blue-shaded band) and individual data points that make up the curve for the period 5400-5100 BC. Vertical lines represent the measurement uncertainties, horizontal bars indicate the number of rings in the samples; in this period individual points of Belfast data represent 20-ring blocks, Seattle 10-ring blocks, and Heidelberg 4-5-ring blocks. Figure produced with the IntCal R package [8].*

Supplementary Note 2

Supplementary Note 2.1

Note on 1 year correction of the Methuselah Walk Bristlecone Pine Chronology

Brehm et al., (2022) showed that the bristlecone pine ^{14}C data preceded the ^{14}C jump at 5259 BCE by 1 year relative to the other tree species used. At the time of publication, the authors had ruled out a dissection or labelling error. The remaining three explanations were that, 1; the rings, although correctly dissected may have been too narrow to achieve a full and perfect separation, 2; the response was caused due to a combination of the timing of the SEP event and the growth season of the tree, or 3; there was a dating error within the bristlecone master chronology.

Matthew Salzer and Christopher Basin conducted extensive testing of the portion of the master bristlecone pine chronology from which samples used in the study were taken. As reported in Brehm et al 2022, the statistics of the sample to the master chronology were excellent (correlation; r = 0.770), however Salzer and Baisan had previously discovered that two changes were required to the older portion of the record which could not be cross-checked against an independent sequence at a different growth location. A correction of 2 years was applied to Wes Ferguson's original 1966 CE dating of this part of the MWK chronology due to an observation by Lamarche and Harlan in 1973 that Ferguson had incorrectly added what he thought were two likely missing rings to his chronology in -2141 and -2680. They suspected that something similar may have happened for the portion of the record between -2680 and -5075 (i.e. impacting the master but not the sample used in the study) as one wrongly inserted ring in this period would explain the off-set observed while still resulting in the strong statistical correlation for the sample. Checking this required physically checking a large number of samples and handwritten records from the 1970s and 1980s which used the skeleton plot method. This work is approaching completion and a candidate error has been identified and confirmed. The results will be published formally, however for the purpose of display in this study there is more than sufficient justification to now display the data in their correct position, especially as this does not impact the main conclusions of this study.

Supplementary Note 2.2

Tree-ring width measurements in Heidelberg format (.fh)

The ring-width measurements below can be copied and pasted in any plain-text editor and saved with a file extension '.fh'. This can then be visualized or analyzed in any dendrochronological software (e.g. dplR library in R). Measurement unit in centimillimeters (0.01 mm).

A plot of these ring-width measurements can be found below, Supplementary Figure 2.1.

HEADER:

Keycode=10611.0

DateEnd=-5155

QualityCode=a

Species=JUSP

Length=123

SapWoodRings=44

Pith=p

WaldKante=3

Location=GRC/Kastoria Dispilio 2019

PersId=

ExcavNr=

CreationDate= 20210107

Dated=dated

DATA:Tree

30	65	74	88	102	95	117	171	108	94
121	58	77	55	75	63	47	46	79	80
92	78	111	129	69	46	40	52	61	38
31	42	35	16	18	36	44	37	23	50
60	67	90	105	88	112	92	78	62	71
76	52	59	96	45	70	71	54	43	67
64	71	68	78	71	54	69	42	42	43
58	57	42	48	43	49	77	36	30	42
18	21	17	21	22	23	26	22	24	29
42	47	29	21	17	17	33	38	27	16
36	44	38	21	14	26	18	10	32	33
24	46	48	46	39	41	20	17	31	23
20	43	53							

HEADER:

Keycode=10206.0

DateEnd=-5153

QualityCode=a

Species=JUSP

Length=142

SapWoodRings=55

Pith=p

WaldKante=3

Location=GRC/Kastoria Dispilio 2019

PersId=

ExcavNr=.

CreationDate= 20210928

Dated=dated

DATA:Tree

66	65	55	54	69	77	106	100	106	101
47	54	15	23	33	41	39	64	68	52
73	58	48	51	57	50	36	41	31	40
39	53	52	50	43	41	44	42	28	34
52	34	43	25	37	46	43	60	66	52
38	50	52	51	46	32	50	56	49	58
66	64	56	52	46	42	44	41	38	51
36	34	47	54	46	40	32	38	39	48
40	53	56	48	34	30	28	44	45	56
56	53	60	69	50	26	14	15	28	37
36	40	28	43	23	23	35	37	45	22
17	21	26	27	51	21	20	50	67	91
55	31	48	35	17	29	31	29	42	56
61	61	66	47	41	42	42	31	55	66
38	48								

HEADER:

Keycode=10070.0

DateEnd=-5240

QualityCode=a

Species=JUSP

Length=58

SapWoodRings=36

Pith=p

WaldKante=3

Location=GRC/Kastoria Dispilio 2019

PersId=

ExcavNr=

CreationDate= 20210928

Dated=dated

DATA:Tree

141 176 146 136 140 106 125 156 152 111

120 131 126 91 114 86 85 66 73 58

144 97 50 87 89 96 80 90 88 59

74 59 69 63 71 60 63 50 43 55

69 40 61 85 50 55 45 74 75 48

73 78 67 54 57 68 54 62

HEADER:

Keycode=10063.0

DateEnd=-5240

QualityCode=a

Species=JUSP

Length=125

SapWoodRings=81

Pith=p

WaldKante=3

Location=GRC/Kastoria Dispilio 2019

PersId=

ExcavNr=

CreationDate= 20210928

Dated=dated

DATA:Tree

90	91	48	102	91	97	61	62	46	62
82	77	91	84	76	60	103	123	97	53
61	70	68	103	80	93	88	116	106	101
112	97	66	94	95	144	103	57	70	69
58	80	71	67	86	65	80	72	70	75
51	33	73	84	82	57	91	62	76	72
66	59	69	83	101	76	79	68	88	65
96	75	91	88	118	142	107	89	61	74
25	39	23	20	26	31	31	97	66	52
83	69	84	77	95	76	44	74	59	63
43	46	35	39	28	57	44	56	25	46
52	35	41	35	66	47	37	50	56	59
63	46	60	69	49					

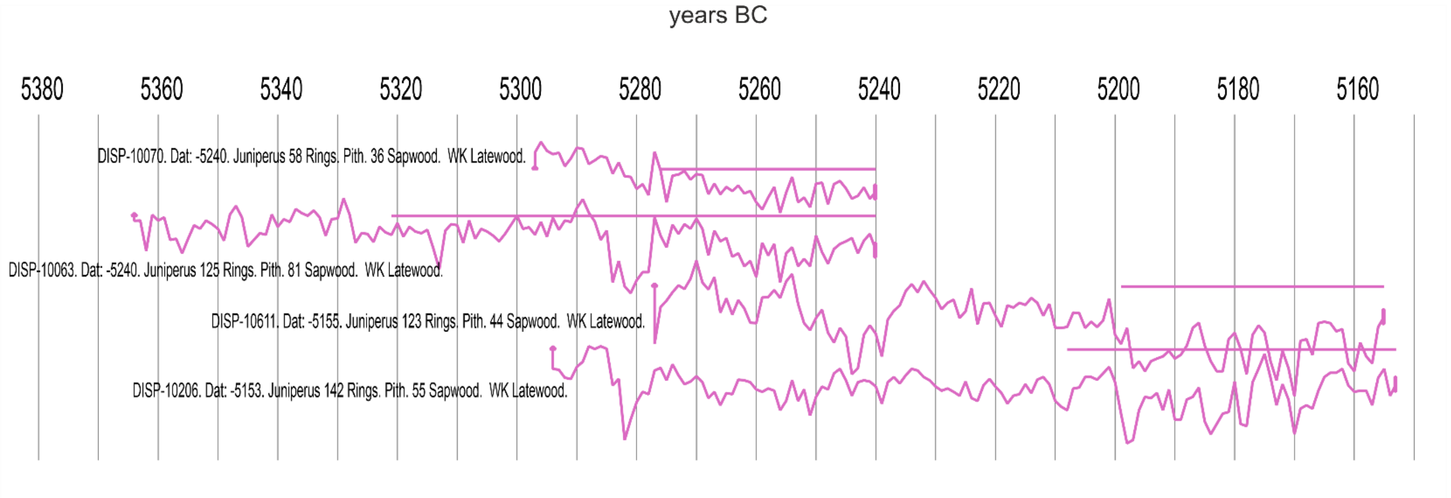

Supplementary Figure 2.1 *Spaghetti plot of the tree-ring width measurements of the wood samples described in the main article text (excavation IDs: DISP-10070, -10063, -10611, -10206) in which the 5259 BC* ^{14}C *event was identified. Measurements go from left (oldest) to right (youngest). Horizontal lines above each sample's ring-width measurements denote the extent of sapwood on that sample. Y-axis scale is semi-logarithmic for each graph; the beginning of each measurement marked by a small triangle denoting the presence of pith; pith triangle and horizontal sapwood line are at 1 mm, so ring-widths below that line are less than 1 mm, ring-widths above the line are above 1 mm.*

Supplementary Information References

1. Hollstein, E. *Mitteleuropäische Eichenchronologie : Trierer dendrochronologische Forschungen zur Archäologie und Kunstgeschichte*. (Mainz am Rhein : von Zabern, 1980).
2. Baillie, M. & Pilcher, J. A Simple Crossdating Program for Tree-Ring Research. *Tree-ring Bull.* 7–14 (1973).
3. Eckstein, D. & Bauch, J. Beitrag zur Rationalisierung eines dendrochronologischen Verfahrens und zur Analyse seiner Aussagesicherheit. *Forstwissenschaftliches Cent.* **88**, 230–250 (1969).
4. Maczkowski, A., Bolliger, M. & Francuz, J. Wetland dendrochronology. An overview of the prehistoric chronologies from the southwestern Balkans. in *Prehistoric Wetland Sites of Southern Europe: Archaeology, Dendrochronology, Palaeoecology and Bioarchaeology.* (eds. Ballmer, A., Hafner, A. & Willy, T.) (Cham: Springer (in press), 2024).
5. Bronk Ramsey, C. Bayesian analysis of radiocarbon dates. *Radiocarbon* **51**, 337–360 (2009).
6. Reimer, P. J. *et al.* The IntCal20 Northern Hemisphere radiocarbon age calibration curve (0–55 cal kBP). *Radiocarbon* **62**, 725–757 (2020).
7. Brehm, N. *et al.* Tree-rings reveal two strong solar proton events in 7176 and 5259 BCE. *Nat. Commun.* **13**, 1–8 (2022).
8. Blaauw, M. IntCal: Radiocarbon Calibration Curves. R package version 0.3.1. https://CRAN.R-project.org/package=IntCal. (2022).
