# Supplementary material for: Absolute dating of the European Neolithic using the 5259 BC rapid 14C excursion
Source: Nat Commun. 2024 May 20;15:4263. doi: 10.1038/s41467-024-48402-1 (PMC11106086; doi:10.1038/s41467-024-48402-1)
Supplement: Supplementary file 3 — Description of Additional Supplementary Materials [file 41467_2024_48402_MOESM3_ESM.pdf]

Description of Additional Supplementary Materials

File Name: Supplementary Data 1

Description: Spreadsheet with all the new raw radiocarbon data presented in the article, the associated uncertainties, and ring numbers.

File Name: Supplementary Data 2

Description: Spreadsheet with averages of the measurements for wood sample DISP-10611 produced at LARA as presented in Fig. 3a.

File name: Supplementary Data 3

Description: Supplementary Data 3 includes the R code and the source data used for the generation of Figures 3 and 5 in the main article text, as well as the OxCal code used for the wiggle-matching of annual 14C in OxCal as presented in Figure 5
