# Supplementary material for: Absolute dating of the European Neolithic using the 5259 BC rapid 14C excursion
Source: Nat Commun. 2024 May 20;15:4263. doi: 10.1038/s41467-024-48402-1 (PMC11106086; doi:10.1038/s41467-024-48402-1)
Supplement: Supplementary file 6 — Reporting Summary [file 41467_2024_48402_MOESM6_ESM.pdf]

nature portfolio

Corresponding author(s): Andrej Maczkowski

Last updated by author(s): Apr 16, 2024

Reporting Summary

Nature Portfolio wishes to improve the reproducibility of the work that we publish. This form provides structure for consistency and transparency in reporting. For further information on Nature Portfolio policies, see our Editorial Policies and the Editorial Policy Checklist.

Statistics

For all statistical analyses, confirm that the following items are present in the figure legend, table legend, main text, or Methods section.

n/a	Confirmed	
☐	☒	The exact sample size (*n*) for each experimental group/condition, given as a discrete number and unit of measurement
☐	☒	A statement on whether measurements were taken from distinct samples or whether the same sample was measured repeatedly
☐	☒	The statistical test(s) used AND whether they are one- or two-sided<br>*Only common tests should be described solely by name; describe more complex techniques in the Methods section.*
☒	☐	A description of all covariates tested
☐	☒	A description of any assumptions or corrections, such as tests of normality and adjustment for multiple comparisons
☐	☒	A full description of the statistical parameters including central tendency (e.g. means) or other basic estimates (e.g. regression coefficient) AND variation (e.g. standard deviation) or associated estimates of uncertainty (e.g. confidence intervals)
☒	☐	For null hypothesis testing, the test statistic (e.g. *F*, *t*, *r*) with confidence intervals, effect sizes, degrees of freedom and *P* value noted<br>*Give P values as exact values whenever suitable.*
☐	☒	For Bayesian analysis, information on the choice of priors and Markov chain Monte Carlo settings
☒	☐	For hierarchical and complex designs, identification of the appropriate level for tests and full reporting of outcomes
☒	☐	Estimates of effect sizes (e.g. Cohen's *d*, Pearson's *r*), indicating how they were calculated

Our web collection on statistics for biologists contains articles on many of the points above.

Software and code

Policy information about availability of computer code

Data collection	The following list of software was used for data collection and analysis:
Data analysis	Dendroplus, v.2013, Ulrich Ruoff, unpublished; DD+ 2.5.4, City of Zurich; CDendro 9.3.1.; Bats 4.30 (Wacker et al., 2010); TRiCYCLE 0.3.1-SNAPSHOT; R 4.0.3 (R Core Team 2020), packages dplr, ggplot2 3.4.4,RColorBrewer 1.3, grid, gridExtra 2.3 ; R Studio; Microsoft Office Suite 2016; OxCal 4.4; QGIS 3.16; Corel DRAW 2019; InkScape 1.0.2-2

For manuscripts utilizing custom algorithms or software that are central to the research but not yet described in published literature, software must be made available to editors and reviewers. We strongly encourage code deposition in a community repository (e.g. GitHub). See the Nature Portfolio guidelines for submitting code & software for further information.

Data

Policy information about availability of data

All manuscripts must include a data availability statement. This statement should provide the following information, where applicable:

- Accession codes, unique identifiers, or web links for publicly available datasets
- A description of any restrictions on data availability
- For clinical datasets or third party data, please ensure that the statement adheres to our policy

All data from this study are available in the article and the Supplementary Information and Supplementary Data 1-3stored on Zenodo repository, doi:10.5281/zenodo.8407222 [https://zenodo.org/records/10981405], for which the use of a DOI resolver is recommended for arriving at the latest version of the data.

Research involving human participants, their data, or biological material

Policy information about studies with human participants or human data. See also policy information about sex, gender (identity/presentation), and sexual orientation and race, ethnicity and racism.

Reporting on sex and gender	N/A
Reporting on race, ethnicity, or other socially relevant groupings	N/A
Population characteristics	N/A
Recruitment	N/A
Ethics oversight	N/A

Note that full information on the approval of the study protocol must also be provided in the manuscript.

Field-specific reporting

Please select the one below that is the best fit for your research. If you are not sure, read the appropriate sections before making your selection.

☐ Life sciences ☐ Behavioural & social sciences ☒ Ecological, evolutionary & environmental sciences

For a reference copy of the document with all sections, see nature.com/documents/nr-reporting-summary-flat.pdf

Ecological, evolutionary & environmental sciences study design

All studies must disclose on these points even when the disclosure is negative.

Study description	The study uses a novel combination of annual radiocarbon and dendrochronology to exactly pinpoint the date of the Mid/Late Neolithic phases at the site of Dispilio, Greece. The recently discovered 5259 BC rapid radiocarbon excursion in dendrochronologyally dated tree-rings from around the Northern Hemisphere enables absolute dating of undated wooden remains with a yearly precision. We use a series of 14C measurements from individual tree-rings to identify the 5259 BC 14C signature.
Research sample	Archaeological wood samples from the prehistoric site of Dispilio, near Kastoria, Northern Greece, representing a sample of 6th millenium BC juniper trees. The waterlogged conditions on the site, which is located next to a lake provided for an excellent preservation of wood whose age i >7000 years. Over 800 wood samples were mapped till 2019, of which 787 where individually analysed for species determination, tree-ring width measurement, cross-dating.
Sampling strategy	As with the vast majority of archaeological material around the world, also in this case the sampling strategy was limited by the extent of the excavation. Almost all mapped wood samples in the Eastern Sector of the excavation were sampled for dendrochornological analysis. Only cross-dated wood samples were sampled for 14C dating from across the tree-ring chronology.
Data collection	Initial description and documentation of the collected wood samples was conduced on-site, using total-station, photogrammetry, Access database and photographic cameras, by the excavation team in Dispilio composed of experienced archaeology professional accompanied by students, some of the authors of the paper also participated in this stage (T.G, J.F., A.M., K.K.). Further processing of samples and ring-width measurements were performed by J.F., A.M., & M.B.. using the tree-ring width measurment software Dendroplus (Ulrich Ruoff, 2013, unpublished). Radiocarbon data was collected using an AMS MICADAS system, see Methods in the main article text.
Timing and spatial scale	Tree-ring width measurements were first conducted on-site in 2019, and continued between late 2020 and early 2022. Radiocabron measurements were performed at various intervals between 2019 and 2023.
Data exclusions	Three 14C measurements from the outer rings of DISP-10063 were excluded due to the small amount of C.<br>Wood samples with obvious growth disturbances and low ring number (>30) were not measured.
Reproducibility	Tree-ring width measurements were collected using standard dendrochronological procedures, under a binocular microscope with a measuring table, by 3 different people located at different places. Radiocarbon measurements were reproduced by two different labs using published methods, see Methods in the main article text
Randomization	Almost all wood remains in the Eastern Sector of the site were sampled, however the sector's location within the site was chosen randomly when excavations began few decades before. The radiocarbon analyses of all tree-ring samples within each lab were treated according to lab protocols, see Methods.
Blinding	The dendrochronological cross-dating was performed without consulting the archaeological plan of the site. Cross-dated samples were only afterwards plotted on the site plan, revealing meaningful groupings.<br>The tree-ring samples sent for 14C measurement to ETH where provided with no information as to where the 5259 BC 14C signature

is located.

Did the study involve field work? ☒ Yes ☐ No

Field work, collection and transport

Field conditions	The excavation is located on the outskirts of the modern village, and about 200 meters away is the Dispilio Excavation Laboratory/ Center. The fieldwork in 2019 was conducted in August/September, when the weather condition were warm with very little rainfall. The conditions for fieldwork in Dispilio provide for a comfortable and safe work.
Location	40.485444 N, 21.289694 E ; h=627 masl
Access & import/export	The site is easily accessible by foot, bike or car.<br>Wood documentation and initial analyses were performed in the Dispilio Excavation Laboratory; due to the Covid-19-related international restrictions on travel and movement of people, sample analysis was continued at the University of Bern, for which a temporary export permit was granted by the Greek Ministry of Culture, ΥΠΠΟΑ/ΓΔΑΠΚ/ΔΣΑΝΜ/ΤΕΕ/Φ77/379195/266411/4122/252, (issued on 24.07.2020)
Disturbance	No specific disturbance was caused by this study. The archaeological site has been intensively excavated since 1992.

Reporting for specific materials, systems and methods

We require information from authors about some types of materials, experimental systems and methods used in many studies. Here, indicate whether each material, system or method listed is relevant to your study. If you are not sure if a list item applies to your research, read the appropriate section before selecting a response.

Materials & experimental systems

n/a	Involved in the study	
☒	☐	Antibodies
☒	☐	Eukaryotic cell lines
☐	☒	Palaeontology and archaeology
☒	☐	Animals and other organisms
☒	☐	Clinical data
☒	☐	Dual use research of concern
☒	☐	Plants

Methods

n/a	Involved in the study	
☒	☐	ChIP-seq
☒	☐	Flow cytometry
☒	☐	MRI-based neuroimaging

Palaeontology and Archaeology

Specimen provenance	Wood specimens were sampled from the prehistoric site of Dispilio, near Kastoria, Northern Greece. 40.485444 N, 21.289694 E.<br>Permit for temporary export of the sampled was issued by the:<br>HELLENIC REPUBLIC<br>MINISTRY OF CULTURE AND SPORTS<br>GENERAL DIRECTORATE OF ANTIQUITIES<br>AND CULTURAL HERITAGE<br>DIRECTORATE OF CONSERVATION OF<br>ANCIENT AND MODERN MONUMENTS<br>DEPARTMENT OF APPLIED RESEARCH, PROTOCOL No.: ΥΠΠΟΑ/ΓΔΑΠΚ/ΔΣΑΝΜ/ΤΕΕ/Φ77/379195/266411/4122/252, (issued on 24.07.2020)
Specimen deposition	The specimens will be permanently deposited at the Dispilio Excavation Laboratory, Dispilio, Greece.
Dating methods	Tree-ring width sequences were cross-dated using standard dendrochronological procedures (see Methods), providing the annual resolution dating within the tree-ring chronology. The tree-ring chronologies were then absolutely dated through radiocarbon dating of individual tree-rings and identification of the radiocarbon spike ("Miyake event") of 5259 BC. Cellulose from the tree-ring samples was extracted following the BABAB method (see Methods and Supplementary Material S3 and Supplementary table T1) at the Laboratory for the Analysis of Radiocarbon with AMS at the University of Bern (LARA) and the Laboratory of Ion Beam Physics at ETH Zürich (ETH), and measured in an AMS MICADAS system.

☒ Tick this box to confirm that the raw and calibrated dates are available in the paper or in Supplementary Information.

Ethics oversight	No ethical approval was required as the trench and the wood were already exposed years ago.

Note that full information on the approval of the study protocol must also be provided in the manuscript.
